# Cang-Ai Volatile Oil Ameliorates Depressive Behavior Induced by Chronic Stress Through IDO-Mediated Tryptophan Degradation Pathway

**DOI:** 10.3389/fpsyt.2021.791991

**Published:** 2021-12-15

**Authors:** Kailing Zhang, Na Lei, Meng Li, Jijun Li, Caijun Li, Yue Shen, Peixin Guo, Lei Xiong, Yuhuan Xie

**Affiliations:** ^1^Basic Medical School, Yunnan University of Chinese Medicine, Kunming, China; ^2^School of Clinical Medicine, Yunnan University of Chinese Medicine, Kunming, China; ^3^Department of Integrative Medicine on Pediatrics, Shanghai Children's Medical Center, Shanghai Jiao Tong University School of Medicine, Shanghai, China; ^4^School of International Education, Yunnan University of Chinese Medicine, Kunming, China; ^5^Ethnic Medical School, Yunnan University of Chinese Medicine, Kunming, China; ^6^Yunnan Provincial University Key Laboratory of Aromatic Chinese Herb Research, Kunming, China; ^7^Yunnan Innovation Team of Application Research on Traditional Chinese Medicine Theory of Disease Prevention at Yunnan University of TCM, Kunming, China

**Keywords:** Cang-ai volatile oil, depression, microglia, IDO, kynurenine pathway, 5-HT

## Abstract

**Background:** Cang-ai volatile oil (CAVO) is a Chinese herbal volatile oil. Previous studies report that CAVO exhibits of anti-depressant and anti-inflammatory effects, and modulates activity of monoamine neurotransmitter. The current study sought to explore whether CAVO exhibits anti-depressant effects of CAVO through inhibition of inflammatory response and regulation of indoleamine 2 and 3-dioxygenase (IDO) mediated tryptophan degradation pathway.

**Methods:** The study established chronic unpredictable mild stress (CUMS) depression-like model using rats. Body weight and food intake of animals were determined, and open field test (OFT), forced swim test (FST), and sucrose preference test (SPT) were performed to explored the behavioral changes of animals. Expression levels of interleukin-6 (IL-6), interleukin-1beta (IL-1β), tumor necrosis factor-alpha (TNF-α), interferon-gamma (IFN-γ), interleukin-4 (IL-4), interleukin-10 (IL-10), kynurenine (KYN), quinolinic acid (QUIN), tryptophan (Trp), kynurenic acid (KYNA), serotonin (5-HT), and 5-hydroxyindole acetic acid (5-HIAA) in the prefrontal cortex of CUMS rats were determined by ELISA. Co-localization of the microglia markers, Iba1 and IL-6 was determined by immunofluorescence. Western blotting was performed to determine the protein expression level of IDO1.

**Results:** The findings of the current study showed that CAVO increased the body weight and food intake of rats and alleviated depression-like behaviors as shown in OFT, FST, and SPT analysis. ELISA assay showed that CAVO decreased IL-6, IL-1β, TNF-α, and IFN-γ levels and increased levels of IL-4 and IL-10 in the prefrontal cortex of CUMS rats. Analysis showed that CAVO significantly reduced KYN and QUIN levels and the ratio of KYN/Trp, whereas it increased the levels of Trp, KYNA, 5-HT, and 5-HIAA. Immunofluorescence analysis showed that CAVO reduced the number of positive cells with co-localization of microglia markers, Iba1 and IL-6. Western blot analysis showed that CAVO decreased the protein expression level of IDO1 in rats.

**Conclusion:** The findings show that the anti-depressant effects of CAVO are mainly attributed to inhibition of the activation of microglia and downregulation of IDO expression, thus inhibiting the kynurenine pathway and reversing the effects exerted on the 5-HT system.

## Introduction

Depression is a mental illness characterized by high morbidity, mortality, and disability (1). Primary clinical symptoms of depression include slow thinking, decreased interest, sleep disturbance, and loss of appetite. WHO reports that more than 350 million people present with major depression globally (2). Treatment of depression is challenging and conventional anti-depressants mainly target monoamine neurotransmitters and are only effective in less than half of the patients (3). Studies report neuro-inflammatory is a key feature of depression, thus studying the anti-inflammatory and anti-depressant mechanisms of potential anti-depressants can help develop more effective anti-depressant drugs (4).

The hypothesis that inflammation is implicated in the pathophysiological process of depression was first proposed in 1991 (4, 5), and several studies report that central nervous system inflammation may contribute to depression. The role of neuro-inflammation in mood disorders has been widely explored (6). Clinical studies report that levels of pro-inflammatory cytokines in the peripheral, cerebrospinal fluid, and hippocampus are significantly higher in patients with depression compared with the levels in health individuals (7, 8). Animal studies present similar findings that expression levels of pro-inflammatory cytokines are increased in rodent depression-like model animals (9). Moreover, pro-inflammatory cytokines can induce depression-like behaviors in rats (10, 11). Studies report that neuronal inflammation is induced by pro-inflammatory cytokines such as interleukin-1β (IL-1β), interleukin-6 (IL-6), and tumor necrosis factor-α (TNF-α). Neuronal inflammation then causes neuroendocrine and neurochemical changes in the brain, leading to occurrence and development of depression (12, 13). Tryptophan (Trp) is an essential amino acid and a substrate for synthesis of human serotonin (5-HT) (13). Tryptophan is converted into other active substances, such as kynuric acid (KYNA) and quinolinic acid (QUIN) through catalysis by the extrahepatic enzymes, indoleamine 2 and 3-dioxygenase (IDO). The first stage of this pathway is catalyzed by the extrahepatic enzyme indoleamine 2 and 3-dioxygenase (IDO) (14). Chronic stress and infection activate IDO and promotes transfer of the available Trp to the kynurenine (KYN) pathway, thus reducing synthesis of 5-HT in the brain (15). Therefore, IDO is plays a significant role as the intersection between inflammation and depression (16). Depression-like behavior can be induced by IDO activation in animals, and application of IDO inhibitors can alleviate depressive state of mice (16, 17). Moreover, clinical studies report consistent findings to those obtained from animal models. Highly expressed activated IDO was observed in plasma and cerebrospinal fluid in patients with suicidal major depression (18). In addition, concentration of QUIN, a metabolite of the kynurenine pathway, is positively correlated with depressive symptoms (19, 20).

Chinese medical records indicate that aromatic herbs have been widely used in treatment of stroke and tumor-related depression for several years (21, 22). Aromatic herbs can activate the brain to alleviate negative emotions of patients, reduce accompanying symptoms, and improve quality of life (23, 24). Therefore, the active ingredients of Chinese aromatic medicine have significant potential in anti-depressant studies (25). Cang-ai volatile oil (CAVO) is a complex preparation of volatile oils extracted from 10 Chinese aromatic herbs, including *Atractylodes lancea* (Thunb.) DC, *Ambrosia artemisiifolia* Linn., *Agastache rugosa* (Fisch & C.A. Mey) Kuntze, *Eupatorium fortunei* Turcz., *Zanthoxylum bungeanum* Maxim., *Amomum kravanh Pierre ex* Gagnep., *Elsholtzia ciliata* (Thunb.) Hyl., and *Syzygium aromaticum* (L.) Merr. & L. M. Perry. The composition of CAVO was detected by gas chromatography-mass spectrometry (GC-MS). The top 10 species of volatile substances in CAVO are Eugenol (42.21%), 1,8-Cineole (11.91%), Patchouli alcohol (9.03%), Acetyl eugenol (8.17%), Linalool (3.66%), Linalyl acetate (3.66%), β-Caryophyllene (1.95%), Terpinen-4-ol (1.95%), Cinene (1.66%), α-Terpineol (1.08%) (26). CAVO is administered through inhalation and has been clinically used for several years for treatment of depressive and physical symptoms and to improve immune response of patients with chronic respiratory diseases by regulating activity of T lymphocytes (27). However, the mechanism underlying activity of CAVO has not been fully elucidated. Preliminary studies report that CAVO regulates DA and 5-HT metabolism in the brains of chronic unpredictable mild stress (CUMS)-induced rats to abrogate their depressive-like behaviors (26, 28). The findings showed that CUMS-induced symptoms of anhedonia are significantly alleviated, and the levels of neurotransmitters in the brain of CUMS model rats are significantly increased. However, the mechanism underlying amelioration of depression-like behaviors has not been fully explored. Studies report that chronic unpredictable stress changes the morphology and activity of neurons in multiple brain areas involved in emotional and cognitive functions (29). In addition, prefrontal cortex (PFC) plays a key role in regulating repeated behavioral changes induced by environmental stress. The current study sought to explore whether CAVO ameliorated depression-like behaviors by inhibiting microglia activation, regulating release of inflammatory factors, downregulating expression of IDO, and increasing 5-HT synthesis in PFC of CUMS rats. The current study established a CUMS-induced depression-like rat models to explore whether the anti-depressant effect of CAVO is associated with inhibition of inflammatory response and regulation of IDO-mediated tryptophan degradation pathway.

## Materials and Methods

### Animals and Treatment

**Figure 1:**
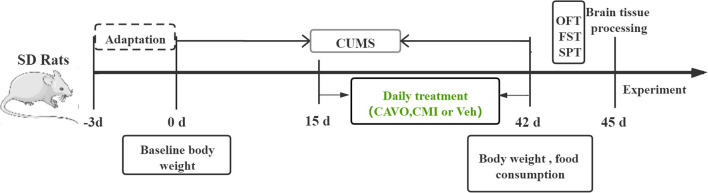
Schematic diagram showing the experimental design of the study.

Male Sprague Dawley (SD) rats (180–220 g) were purchased from Chengdu Dashuo Experimental Animal Co., Ltd., with the license number: SCXK (Chuan) 2015-030 and quality inspection certificate number: 51203500007196. Rats were raised in SPF-level animal observation rooms under controlled temperature (24 ± 1°C) and humidity (50 ± 10%) with a 12-h light-dark cycle. Rats were acclimatized through 3 days of adaptive feeding, then randomly assigned 4 groups (*n* = 8), namely control, model, clomipramine, and CAVO groups. 20 mg/kg/d clomipramine was administered by gavage, whereas 14.6 μg/kg/d CAVO [effective concentration proved by previous experiments (28)] and an equivalent volume of distilled water and (for model and control groups) were administered by inhalation from the 15th to 42nd day of CUMS modeling (Figure 1). CAVO or distilled water was added to a nebulizer and nebulized for 10 min for per rat. All rat experiments were approved by the Animal Ethics Experiment Committee of Yunnan University of Traditional Chinese Medicine (approval number: R-06202092) and were performed in accordance to *the Guidelines for the Care and Use of Laboratory Animals* published by the National Institute of the United States.

### Experimental Drugs

Preparation of CAVO and analysis using high-performance liquid chromatography to identify and quantify contents in CAVO were performed as described previously (26). CAVO solution was prepared by dissolving CAVO in double-distilled water and Tween-80 at a ratio of 0.15% and the mixture was stored in a refrigerator at 4°C for further use. The positive control drug, clomipramine hydrochloride tablets (batch number: 20180604) was purchased from Jiangsu Enhua Pharmaceutical Co., Ltd.

### Establishment of a CUMS Model

The rats in the control group were housed in group, and the rats in other groups were housed separately. The following 7 stress stimuli were randomly induced during established of the 42-day model as described previously [(30); Tables 1, 2]. The feeding of the control group was not interfered with except for water deprivation for 24 h before the sucrose preference test (SPT). Notably, irregular pressure stimulation should be used when establishing the CUMS models to avoid adjustment of rats to pressure stimulation.

**Table 1:** CUMS procedure.

**Stressors**	**Duration**
Inversion of light/dark cycle	24 h
Cage tilting (without bedding)	12 h
Tail nip (0.5–1 cm from the end of the tail)	1 min
Hot water swimming (45°C)	5 min
Wet bedding (300 mL of water per individual cage to make the bedding wet)	24 h
Food and water deprivation	24 h

**Table 2:** CUMS schedule.

**Weekly**	**Monday**	**Tuesday**	**Wednesday**	**Thursday**	**Friday**	**Saturday**	**Sunday**
First week	Inversion of light/dark cycle	Tail nip	Hot water swimming (45°C)	Wet bedding	Cage tilting	Cold water swimming (4°C)	Food and water deprivation
Second week	Tail nip	Inversion of light/dark cycle	Hot water swimming (45°C)	Cage tilting	Food and water deprivation	Cold water swimming (4°C)	Wet bedding
Third week	Cage tilting	Hot water swimming (45°C)	Wet bedding	Inversion of light/dark cycle	Cold water swimming (4°C)	Tail nip	Food and water deprivation
Forth week	Wet bedding	Hot water swimming (45°C)	Inversion of light/dark cycle	Cage tilting	Tail nip	Food and water deprivation	Cold water swimming (4°C)
Fifth week	Inversion of light/dark cycle	Wet bedding	Hot water swimming (45°C)	Cage tilting	Food and water deprivation	Tail nip	Cold water swimming (4°C)
Sixth week	Tail nip	Cold water swimming (4°C)	Wet bedding	Inversion of light/dark cycle	Cage tilting	Food and water deprivation	Hot water swimming (45°C), food intake

### Body Weight and Food Intake

All rats were weighed before establishment of the model to obtain the baseline body weight, and changes in body weight were determined by subtracting the baseline weight from the bodyweight at week 6. Each group was deprived of food for 24 h before food intake was determined. Rats were fed with 50 g of food at 8 a.m. on 42nd day after food deprivation. At 8 a.m. the next day, the remaining food was weighed, and food intake was calculated as shown below:


$$\begin{array}{l} \text{Food intake }\left(\text{g}\right)=50\text{g}-\text{ remaining food }\left(\text{g}\right)\\ \text{Body weight }\left(\text{g}\right)=\text{week}-6\text{ body weight }\left(\text{g}\right)\\ \;-\text{Baseline body weight }\left(\text{g}\right) \end{array}$$

### Open Field Test (OFT)

The OFT was done at 10 a.m. on the 43rd day. An open field box and software (XR-XZ301) (Shanghai New Software Information Technology Co., Ltd.) were used for OFT. The method described previously (31). Rats were gently placed at the center of the open field box (100 × 100 × 40 cm), and the total moving distance and number of erections of the rat in 5 min were tracked and recorded using the software. The open field box was wiped with 75% alcohol and cleaned after each animal was tested to prevent the residual odor from affecting the test results of the next animal.

### Sucrose Preference Test (SPT)

The SPT was done at 10 a.m. on the 44th day. Rats were deprived of water for 24 h prior to conducting SPT (32). The water bottle was then replaced by two bottles, one containing 1% sucrose and the other pure water, with the weight of each determined and recorded. Rats were allowed to freely choose either of the two water bottles to drink. The position of the water bottles was changed every 12 h, and were removed after 24 h. The liquid intake of the two bottles was then weighed and recorded to determine the sucrose preference rate, using the formula below:


$$\begin{array}{l} \text{Sucrose preference rate }\left(\text{\%}\right)\\ =\left[\text{sucrose intake }\left(\text{g}\right)/\text{total drinking water }\left(\text{g}\right)\right]\times \;100\% \end{array}$$

### Forced Swim Test (FST)

The FST was done at 10 a.m. on the 45th day. Forced swim equipment and analysis software (DOiT02-FS-G) (Shanghai New Software Information Technology Co., Ltd.) were used for FST. Rats were acclimatized to swimming for 15 min in a plexiglass cylinder with a water depth of 30 cm at 25°C on the day before the test (33). On the test day, rats were placed in the cylinder with a water depth of 30 cm at 25°C for 10 min, and the immobility time of rats was calculated during the last 8 min. The time spent floating on water was recorded as the stationary time.

### Enzyme-Linked Immunosorbent Assay (ELISA)

After euthanization, prefrontal cortex tissues were quickly separated on ice. After the RIPA lysate was added, the tissue was ground to obtain the supernatant, which was stored in −80°C refrigerator for later use. Levels of Trp, KYN, QUIN, KYNA, 5-HT, 5-hydroxyindole acetic acid (5-HIAA), IL-6, IL-1β, TNF-α, and IFN-γ in the supernatant of tissue homogenate were determined using commercial ELISA kits (Shanghai Jining) according to the manufacturer's instructions. The optical density (OD) of each well was determined using an ELISA reader at 450 nm.

### Immunofluorescence Analysis

Rats were anesthetized and the brains were perfused with pre-cooled saline and 4% paraformaldehyde. The brain tissue was then harvested and fixed with 4% paraformaldehyde overnight. Tissues were dehydrated with 15 and 30% sucrose solution, then embedded on OCT and stored under refrigeration at −80°C. For immunofluorescence staining, samples were retrieved from the refrigerator, re-warmed for 30 min, and rinsed with PBS for 10 min. Samples were then sealed using a blocking solution for 1 h at room temperature. Samples were then incubated with primary antibody (Iba1:1:200, Abcam; IL-6:1:200, GeneTex) overnight at 4°C. Samples were washed with PBS 3 times for 10 min after incubation. Further, samples were incubated with secondary antibodies labeled with Alexa fluor 488 and Alexa fluor 647 at room temperature for 1 h. After incubation, samples were rinsed thrice with water, then the slices were incubated with DAPI for 5 min at room temperature and stored in the dark. Imaging was performed using a confocal microscope (Nikon, Japan).

### Western Blotting

Total protein was extracted using RIPA lysate buffer and quantified using BCA kit (Beyotime Biotechnology). Proteins were separated by 12% SDS-PAGE and transferred onto PVDF membranes (Millipore, USA). Membranes were blocked with 5% skim milk at room temperature for 1 h, then incubated with rabbit anti-IDO1 (1:1,000, Proteintech) and rabbit anti-GAPDH (1:10,000, Proteintech) overnight at 4°C. Membranes were further incubated with secondary antibodies (1:10,000, proteintech) for 1 h. Detection was performed using iBright FL1500 (Thermo fisher, USA).

### Statistical Analysis

Data were presented as Mean ± SEM and analyzed using one-way ANOVA with Tukey test (homogeneous variables) or Games-Howell (heterogeneous variables). *P* < 0.05 indicated that the difference was statistically significant.

## Results

### Effect of CAVO on CUMS-Induced Depression-Like Behavior

**Figure 2:**
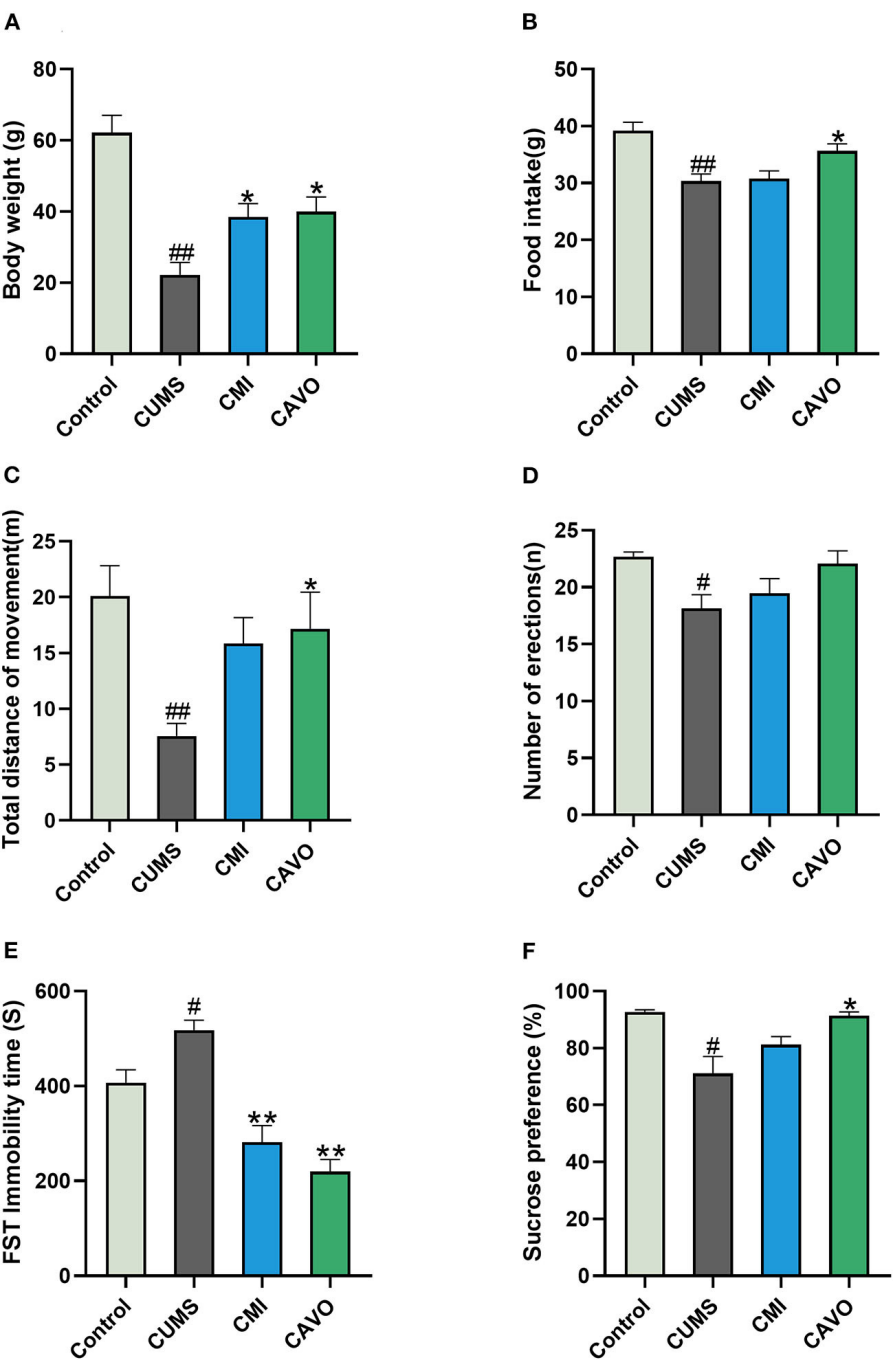
Effect of CAVO on CUMS-induced depression-like behavior. **(A)** Body weight gain of rats at week 6. **(B)** Food intake of rats in the week 6. **(C)** Total distance covered in the OFT. **(D)** Number of erections in the OFT. **(E)** Immobility time in FST. **(F)** Sucrose preference rate of rats in SPT (*n* = 8, means ± SEM) (^#^*P* < 0.05 and ^##^*P* < 0.01 vs. the control group. *P < 0.05 and **P < 0.01 vs. the model group).

The current study used body weight gain, food intake, OFT, FST, and SPT to evaluate the success of the CUMS model and the anti-depressant effect of CAVO. The findings showed that weight gain and food intake of rats in the model group were lower compared with that of rats in the control group (*P* < 0.01, Figure 2). Analysis showed that CAVO treatment resulted in increase in body weight and food intake (*P* < 0.05). The OFT total distance covered by the rats in the model group was significantly lower after 6 weeks of CUMS modeling compared with that of the blank control group (*P* < 0.01). In addition, the number of standing times was significantly reduced compared with that of the control group (*P* < 0.05). The total distance covered by rats in the CAVO and clomipramine groups were significantly higher compared with the distance covered by rats in the model group (*P* < 0.05). Moreover, the number of standing times in the CAVO group had a rising trend compared with the distance covered by rats in the model group. FST test showed that rats in the model group stayed immobile for longer durations compared with the duration for rats in the control group (*P* < 0.05). Immobility time was significantly less for rats in CAVO and clomipramine group compared with the immobility time for rats in the model group (*P* < 0.01). The findings showed that sucrose preference rate for rats in the model group was significantly lower compared with those in the control group (*P* < 0.05). However, the sucrose preference rate increased significantly after 4 weeks of CAVO administration (*P* < 0.05). These findings indicate that CAVO improved depression-like behavior induced by CUMS in rats.

### Effect of CAVO on the Levels of Cytokines in the Prefrontal Cortex of CUMS Model Rats

**Figure 3:**
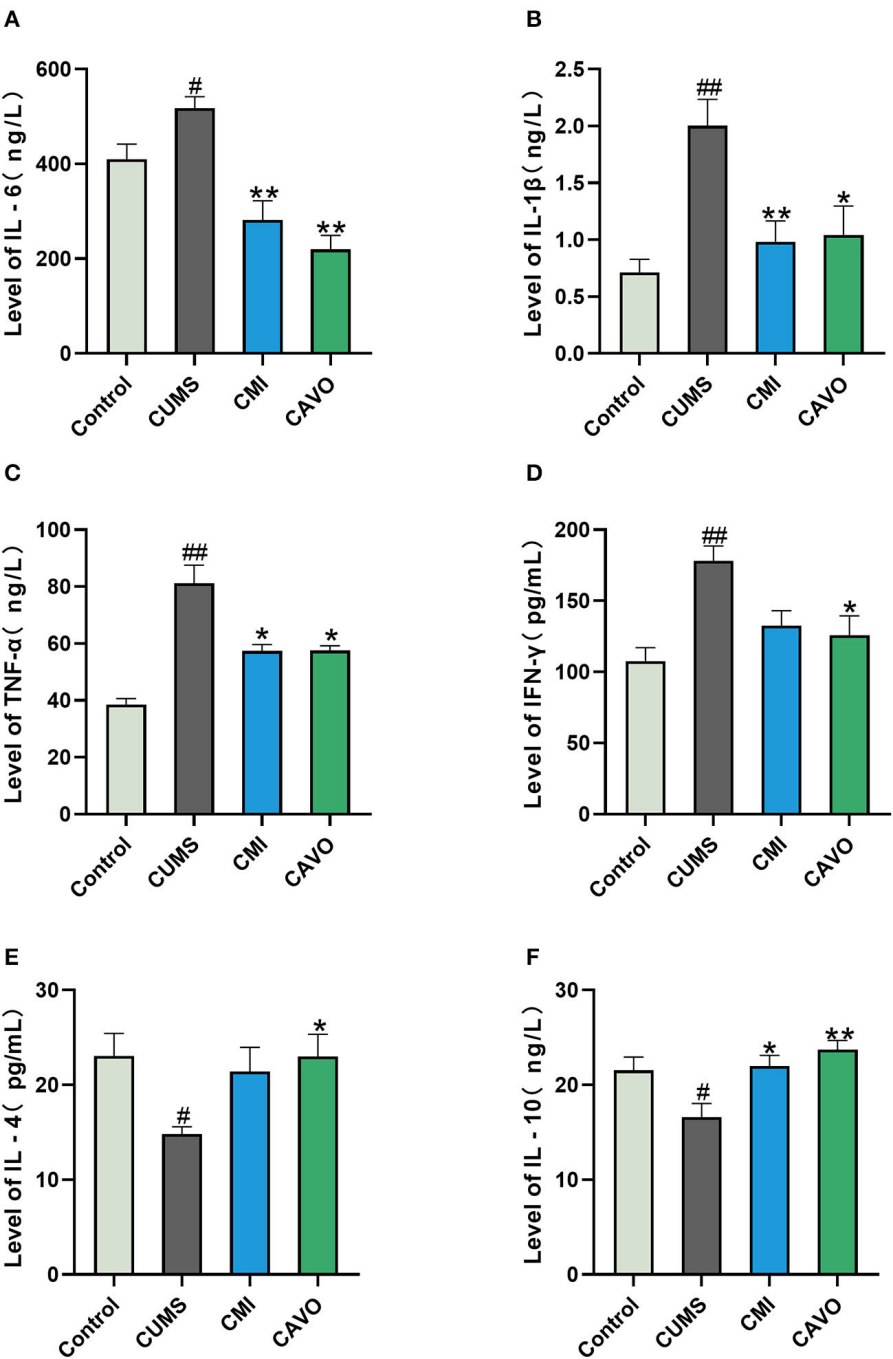
Effect of CAVO on expression levels of cytokines in the prefrontal cortex of CUMS model rats. **(A)** Level of IL-6 in the prefrontal cortex of rats. **(B)** Level of IL-1β in the prefrontal cortex of rats. **(C)** Level of TNF-α in the prefrontal cortex of rats. **(D)** Level of IFN-γ in the prefrontal cortex of rats. **(E)** Level of IL-4 in the prefrontal cortex of rats. **(F)** Level of IL-10 in the prefrontal cortex of rats (*n* = 8, means ± SEM) (^#^*P* < 0.05 and ^##^*P* < 0.01 vs. the control group. *P < 0.05 and **P < 0.01 vs. the model group).

The findings showed that the expression levels of IL-6, IL-1β, TNF-α, and IFN-γ in the prefrontal cortex of rats in the control group was significantly low compared with the level of cytokines in the prefrontal cortex of CUMS rats (*P* < 0.05, Figure 3). On the contrary, low expression levels of IL-6, IL-1β, TNF-α, and IFN-γ were observed in the prefrontal cortex of rats in the CAVO or clomipramine treatment group compared with the levels in rats in the control group (*P* < 0.01 and *P* < 0.05, respectively). Expression level of IL-4 and IL-10 in the prefrontal cortex of rats in the model group were lower compared with the levels in the control group (*P* < 0.05). Notably, CAVO treatment significantly increased the expression levels of IL-4 and IL-10 in the prefrontal cortex of rats in the model group compared with the expression levels of rats in the control group (*P* < 0.01 and *P* < 0.05, respectively).

### Effect of CAVO on Co-expression of Iba1 and IL-6 in the Prefrontal Cortex of CUMS Model Rats

**Figure 4:**
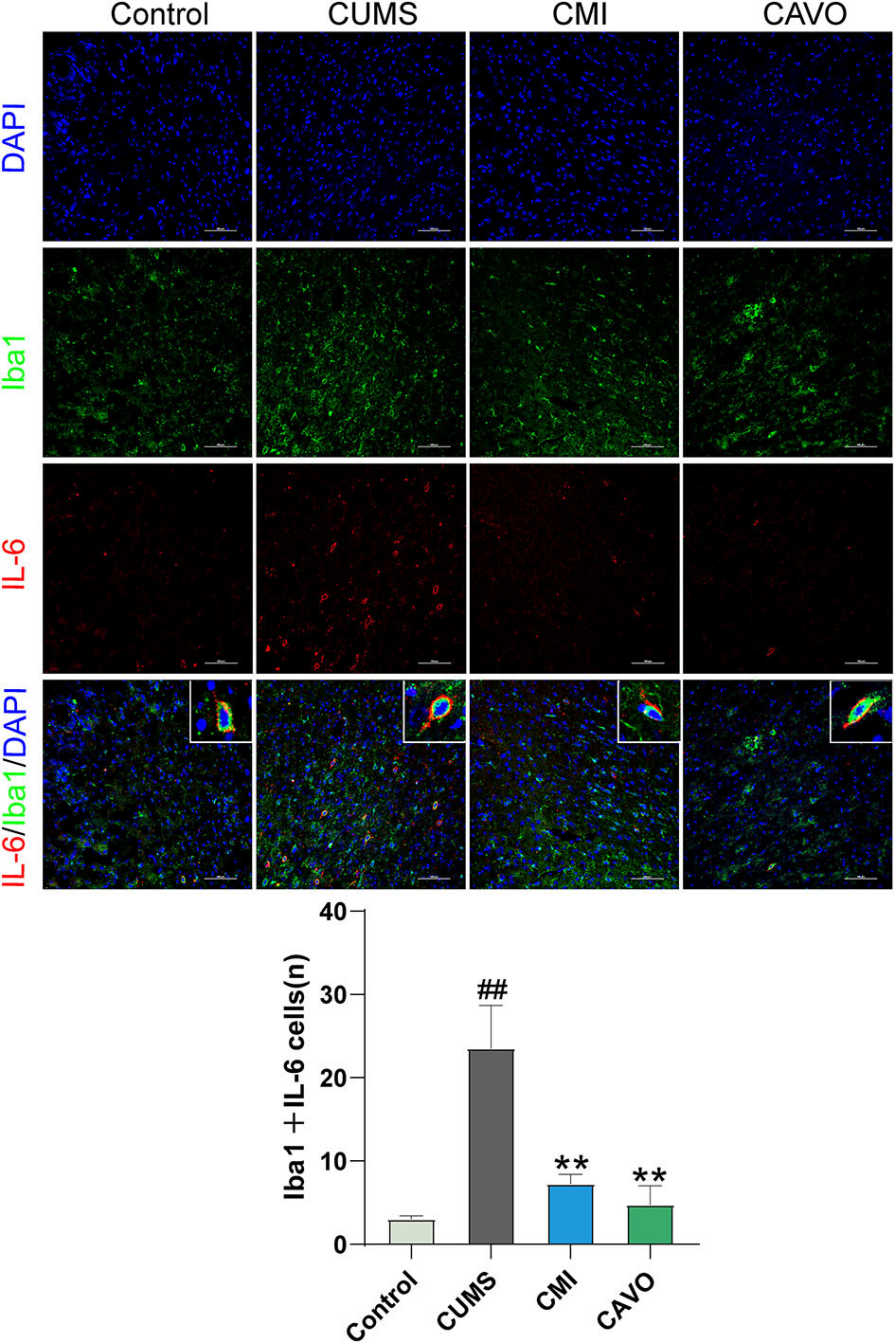
Effect of CAVO on co-expression of Iba1 and IL-6 in the prefrontal cortex of CUMS model rats. DAPI was used as a marker for the nucleus, AF488 for Iba-1, and CY3 for IL-6 (*n* = 4, means ± SEM, ×200 magnification) (^#^*P* < 0.05 and ^##^*P* < 0.01 vs. the control group. *P < 0.05 and **P < 0.01 vs. the model group).

The findings showed that rats in the CUMS model group had significantly higher number of Iba1 and IL-6 co-localized positive cells in the prefrontal cortex compared with the number of Iba1 and IL-6 co-localized positive cells in the control group (*P* < 0.01, Figure 4). The number of Iba-1 and IL-6 co-localized positive cells in the frontal cortex of rats in the CAVO and clomipramine groups were significantly low compared with the number of co-localized cells in the model group (*P* < 0.01).

### Effect of CAVO on IDO1 Protein Expression in the Prefrontal Cortex of CUMS Model Rats

**Figure 5:**
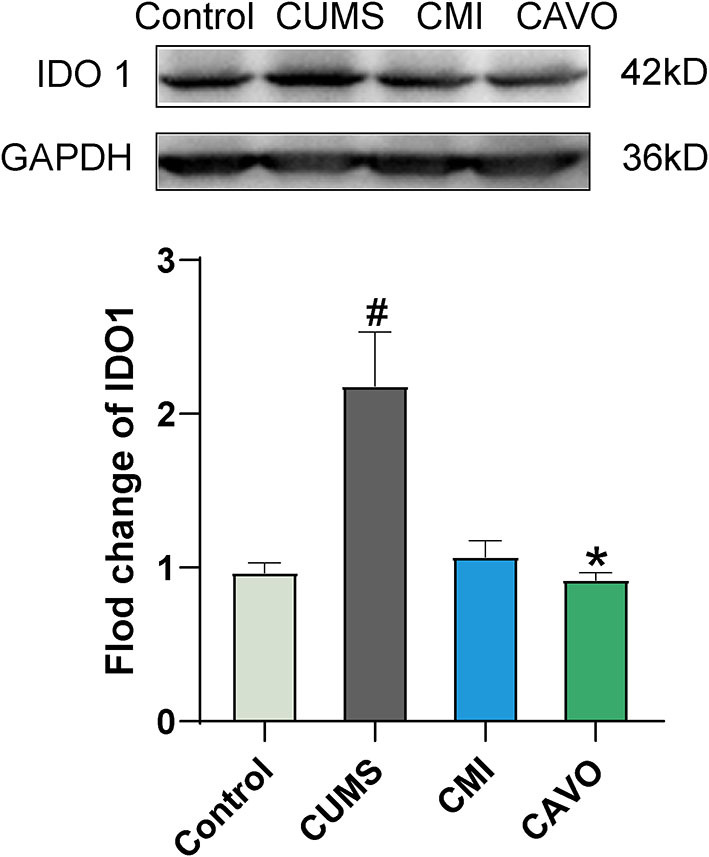
Effect of CAVO on IDO1 protein expression level in the prefrontal cortex (*n* = 8, means ± SEM) (^#^*P* < 0.05 and ^##^*P* < 0.01 vs. the control group. *P < 0.05 and **P < 0.01 vs. the model group).

Release of inflammatory cytokines can lead to IDO1 activation, thus the level of IDO1 was determined. The findings showed that CUMS model rats had significantly higher IDO1 protein expression levels in the prefrontal cortex compared with the levels in rats in the control group (*P* < 0.05, Figure 5). Expression level of IDO1 was significantly downregulated in rats administered with CAVO compared specified with the expression level in rats in the model group (*P* < 0.05).

### Effect of CAVO on Metabolites Involved in Kynurenine Pathway in the Prefrontal Cortex

**Figure 6:**
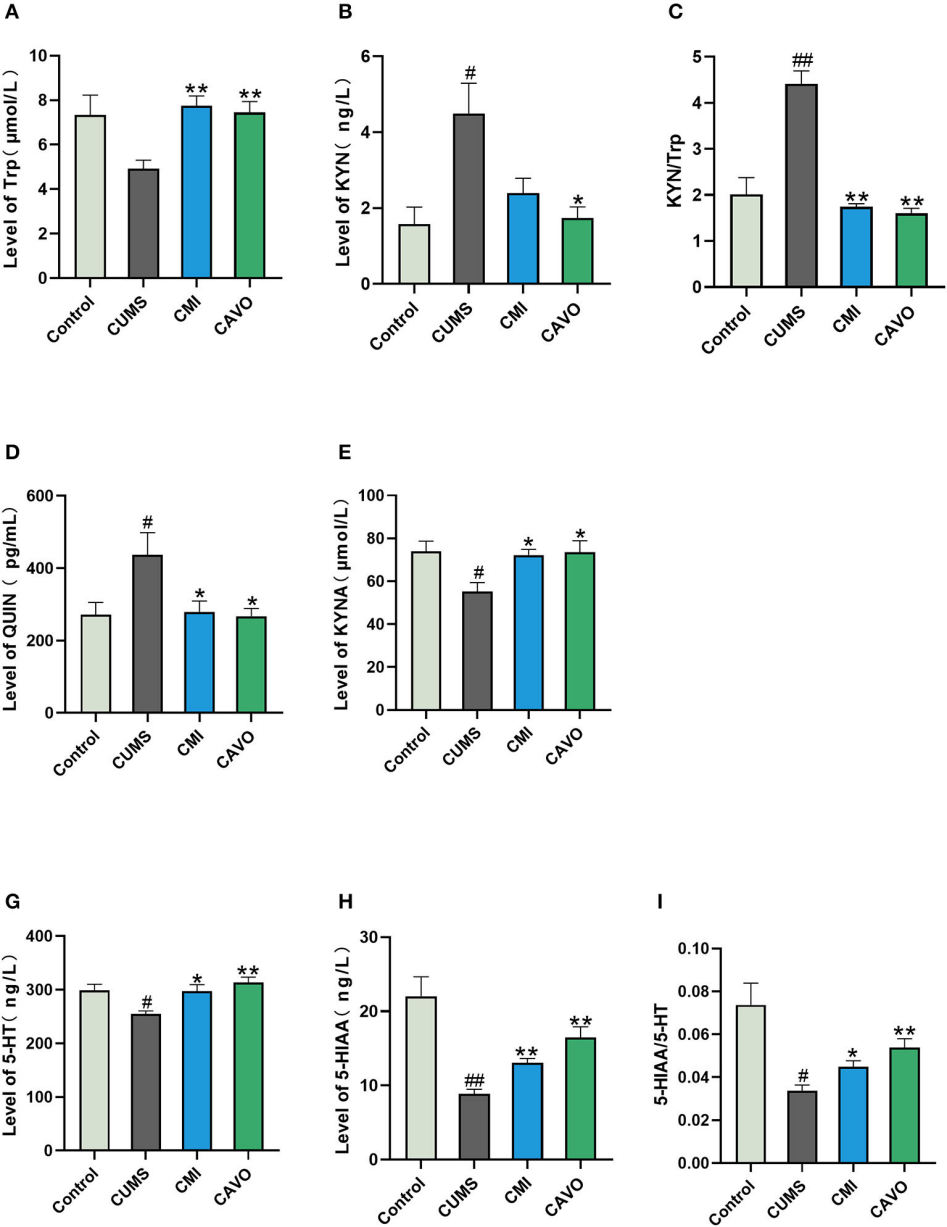
Effect of CAVO on level of metabolites involved in kynurenine pathway in prefrontal cortex. **(A)** Level of tryptophan (Trp). **(B)** Level of kynurenine (KYN). **(C)** Ratio of KYN/Trp. **(D)** Level of quinolinic acid (QUIN). **(E)** Level of kynurenic acid (KYNA). **(F)** Level of serotonin (5-HT). **(G)** Level of 5-hydroxyindole acetic acid (5-HIAA) (*n* = 8, means ± SEM) (^#^*P* < 0.05 and ^##^*P* < 0.01 vs. the control group. *P < 0.05 and **P < 0.01 vs. the model group).

IDO activation accelerates metabolism of tryptophan in the kynurenine pathway, leading to a decrease in tryptophan content. This limits synthesis of 5-HT and results in an insufficient content of 5-HT in the brain. In addition, IDO can inhibit metabolism of kynurenine to kynurenic acid and promote metabolism of quinolinic acid. Therefore, changes in the levels of Trp, KYN, QUIN, KYNA, 5-HT, 5-HIAA, and Trp in the prefrontal cortex of rats were explored. The findings showed that CUMS significantly suppressed the levels of KYNA, 5-HT, and 5-HIAA in rats in the model group compared with the levels in rats in the control group (*P* < 0.05 and *P* < 0.01, respectively; Figure 6). Treatment with CAVO and clomipramine significantly reversed these changes (*P* < 0.05 and *P* < 0.01, respectively). In addition, there exists a significant increase in Trp and a decrease in the ratio of KYN/Trp in therapy groups (*P* < 0.01). Notably, CUMS significantly increased the levels of KYN and QUIN in rats in the model group as compared to control group (*P* < 0.05 and *P* < 0.01, respectively). Meanwhile, CAVO and clomipramine therapy showed a significant alteration of these metabolites relative to model. (*P* < 0.05 and *P* < 0.01, respectively). Consistent with the results for 5-HT and 5-HIAA, the 5-HIAA/5-HT ratio was also reduced in the CUMS group compared to the control group (*P* < 0.05 and *P* < 0.01, respectively), while CAVO treatment significantly reversed this ratio (*P* < 0.01).

## Discussion

The current study explored the antidepressant-like effects of CAVO on CUMS-induced depression-like model rats and the possible underlying mechanisms. CUMS rat model was successfully established through subjecting rats to continuous mild unpredictable stress for 6 weeks. Food intake, weight gain, SPT, and OFT were used to evaluate whether the CUMS model was established successfully. The findings showed that rats in the CUMS model group exhibited a significant decrease in food intake, body weight gain, sucrose preference rate, total distance of movement, and the number of erections observed during OFT. However, FST analysis showed an increase in the immobility time of rats in the model group. These behaviors are similar to the core symptoms exhibited by most depression patients. The findings showed that 2-week CAVO treatment significantly increased food intake, body weight gain, sucrose preference rate, total distance of movement, and the number of erections. In addition, administration of CAVO decreased the immobility time observed in FST, whereas clomipramine showed no effect on CUMS-induced decrease in food intake, body weight gain, and number of erections observed in OFT. Previous studies reported that CAVO can exhibits anti-depressant effects in various animal models of depression which is consistent with findings of the current study (26). The effects of the compounds in CAVO on expression levels of inflammatory cytokines were determined to further explore the mechanism of CAVO in rats. Eugenol is implicated in improving depressive behaviors (34), alleviates inflammation in damaged spinal cord and lowers oxidative stress and reduces the levels of neural apoptosis-associated molecules (35, 36). Linalool inhibits production of TNF-α, IL-1β, nitric oxide (NO), prostaglandin E2 (PGE2) in LPS-induced microglia in a dose-dependent manner (37–39). 1,8-cineole exhibits strong anti-inflammatory, anti-oxidant effects, and anti-atherosclerotic activity in various inflammation models *in vivo* and *in vitro* (40–42). The anti-inflammatory effects of these compounds present in CAVO provides a basis for further study of the anti-depressant mechanism of CAVO.

Microglia are resident macrophages of the central nervous system (CNS), which are characterized by the membrane surface marker, Iba1 (43). Microglia play a key role in the immune function of the brain (44). Resting microglia are activated to adopt the M1 state and the alternatively activated (M2) state in response to various stimulations (45). M1 phenotype of microglia promotes release of pro-inflammatory mediators including IL-1β, IL-6, TNF-α, and IFN-γ, which induce inflammatory process (46). On the contrary, the M2 phenotype of microglia promotes phagocytosis and release of anti-inflammatory cytokines, IL-10, and IL-4, thus reducing inflammation and ultimately protects neurons (43, 44, 47). Transient neuro-inflammation promotes fight against pathogenic microorganisms and removal of cellular debris released from injured or dead cells (46). However, excessive long-lasting activation of microglia and neuro-inflammatory disorders can lead to death of neuronal cells (48). The findings of the current study showed an increase in the level of inflammatory cytokine factors including IL-6, IL-1β, TNF-α, and IFN-γ in the PFC of model rats whereas the levels of IL-4 and IL-10 were reduced. In addition, the findings indicated microglial activation as shown by increase in co-expression of Iba1 and IL-6 in PFC microglia. These findings are consistent with findings reported by previous studies (49). Notably, these changes were significantly abrogated by CAVO treatment, and the symptoms of depressed rats were alleviated. The control drug clomipramine showed similar effects on reduction of the level of inflammatory factors and inhibition of microglial activation to the effects by CAVO, implying that anti-depressants exhibit their activity through anti-inflammatory effects (50–52).

Cytokines secreted by activated microglia further promote differentiation of microglia into M1 state (53). Long-term pro-inflammatory state of M1 microglia disrupts their ability to perform proper phagocytosis and respond to anti-inflammatory signals. Pro-inflammatory cytokines, mainly IFN-γ, can activate IDO, a key enzyme that metabolizes Trp in the kynurenine pathway (54). IDO is a complex comprising two homologous proteins IDO1 and IDO2, with similar structure and function (55). IDO1 is a tryptophan degrading enzyme that metabolizes tryptophan into kynurenine through the kynurenine pathway, resulting in insufficient raw materials for production of 5-HT, and ultimately inhibits 5-HT synthesis (56–58). Although, IDO2 has similar structure and function to IDO1, its biological mechanism has not been fully elucidated (55). Kynurenine pathway is present in macrophages and microglial cells and partly in astrocytes (59). The rate-limiting step of the KYN pathway in the brain is the step involving conversion of Trp to KYN (60). KYN can subsequently be metabolized in two distinct routes: either by kynurenine monooxygenase, kynurinase, and 3-hydroxy-o-aminobenzoic acid 3 and 4-dioxygenase to produce QUIN in microglia and macrophages, or by kynurenine aminotransferase to produce KYNA in astrocytes (61). KYNA and QUIN act as weak NMDA receptor antagonist and agonist, respectively (61, 62). Western blot analysis in the current study showed that the expression level of IDO was significantly increased in the CUMS model group compared with the control group. Level of Trp in the CUMS group was significantly lower, whereas the levels of KYN and QUIN were significantly higher compared with the levels in the control group. Therefore, the KYN/Trp ratio which is an effective marker for immune-mediated IDO activation, showed significant increase in rats in the CUMS model group.

The findings showed a significant decrease in the level of KYNA, 5-HT, and its metabolite 5-HIAA in the CUMS group compared with the level in the control group. These findings indicate that increase in chronic stress induced release of inflammatory cytokines that activated IDO in microglia and caused preferential activation of the QUIN pathway over the 5-HT pathway. This implies that serotonin depletion and imbalance between neuro-toxic and neuroprotective metabolites may be the main drivers of depression. CAVO downregulated the Trp-metabolizing enzyme IDO, which subsequently inhibited depletion of Trp and 5-HT and modulated the balance of Trp catabolites.

In conclusion, anti-depressant effects of CAVO are mainly through inhibiting activation of microglia and downregulating expression of IDO, thus inhibiting the kynurenine pathway and reversing the effects of the 5-HT system. These findings provide a potential treatment basis for clinical application of CAVO for management of depression. In addition, the findings indicate that CAVO can be used as an alternative drug for treatment and prevention of depression through the kynurenine pathway.

## Data Availability Statement

The original contributions presented in the study are included in the article/supplementary material, further inquiries can be directed to the corresponding authors.

## Ethics Statement

The animal study was reviewed and approved by Animal Experimental Ethics Committee of Yunnan University of Chinese Medicine.

## Author Contributions

YX and PG conceived and designed the study. KZ and ML performed experimental analysis. NL and CL were involved in the experimental work and data analysis. KZ wrote the first draft of the manuscript. YX, LX, and NL critically revised the manuscript. YS improved the language the manuscript. All authors have significantly participated in the manuscript preparation, were involved in manuscript writing, and approved the final version of the manuscript.

## Funding

This study was financially supported by the National Natural Science Foundation of China (NSFC) (Nos. 82060823, 82074421, and 81560740) and Natural Science Foundation of Yunnan Province (Nos. 2017FA045 and 202001ZA070001-033).

## Conflict of Interest

The authors declare that the research was conducted in the absence of any commercial or financial relationships that could be construed as a potential conflict of interest.

## Publisher's Note

All claims expressed in this article are solely those of the authors and do not necessarily represent those of their affiliated organizations, or those of the publisher, the editors and the reviewers. Any product that may be evaluated in this article, or claim that may be made by its manufacturer, is not guaranteed or endorsed by the publisher.

## Abbreviations

5-HT: serotonin
5-HIAA: 5-hydroxyindole acetic acid
CAVO: Cang-ai volatile oil
CUMS: Chronic Unpredictable Mild Stress
FST: forced swim test
IDO: indoleamine-2,3-dioxygenase
IFN-γ: interferon-gamma
IL-4: interleukin-4
IL-6: interleukin-6
IL-1β: interleukin-1beta
Iba1: ionized calcium binding adaptor molecule 1
KYN: kynurenine
KYNA: kynurenic acid
NF-κB: nuclear factor kappa-B
NO: nitric oxide
NMDA: N-methyl-D-aspartate
OFT: open field test
PGE2: prostaglandin E2
QUIN: quinolinic acid
SPT: sucrose preference test
TNF-α: tumor necrosis factor-alpha
Trp: tryptophan.
